# 67 SGLT2 Inhibitor Therapy for Burn-Induced Mitochondrial Dysfunctions

**DOI:** 10.1093/jbcr/iraf019.067

**Published:** 2025-04-01

**Authors:** Hiroki Ogata, Hiroyuki Morinaga, Yoh Sugawara, Nobuo Yasuda, Sora Kikuchi, Erica Yasuhara, Asahi Adachi, Alyssa Yasuhara, Jeevendra Martyn, Shingo Yasuhara

**Affiliations:** Shriners Children’s - Boston; Shriners Children’s - Boston; Shriners Children’s - Boston; Okayama Healthcare Professional University; Boston University; Wellesley College; Shriners Children’s - Boston; Shriners Children’s - Boston; Shriners Children’s - Boston; Shriners Children’s - Boston

## Abstract

**Introduction:**

Mitophagy, the autophagic process targeting mitochondria, serves as a vital cellular and mitochondrial quality control (QC) system, implicated in various human diseases including burn-induced skeletal muscle mitochondrial dysfunctions. SGLT2 inhibitors (SGLT2Is), such as empagliflozin (EMPA), were originally designed to manage type 2 diabetes. SGLT2Is confer their advantages even in some organs where SGLT2 expressing is lacking, suggesting their efficacy via non-canonical pathways. Here in this study, we examined the therapeutic effect of EMPA on burn-induced mitophagy defect in myocytes.

**Methods:**

We engineered C2C12 cells expressing photoconvertible fluorescent protein mito-Kaede or the pH-sensitive fluorescent marker tandem-fluorescent LC3 (tf-LC3). Control cells and those subjected to 24 hours of burn serum treatment underwent mitochondrial stress induction with carbonyl cyanide chlorophenylhydrazone (CCCP) on the day of analysis to trigger the mitophagy response. Subsequently, we monitored mitophagy flux (mito-Kaede) or the progression of autophagosome maturation. To evaluate whether SGLT2 inhibition mitigates mitophagy defects caused by burn serum stress, control and burn groups, with or without EMPA treatment, were subjected to acute stress by incubating with 5 μM CCCP. Mitochondrial production of reactive oxygen species (ROS) was assessed by CellROX staining.

**Results:**

The mitophagy flux assay employing mito-Kaede revealed a significant disruption in the mitophagy response to CCCP-induced stress in burn serum-treated cells, with a notable reduction (control vs. burn = 35.7% vs.11.3% the higher the value, the better the response). Concurrently, monitoring of CCCP-induced autophagy/mitophagy maturation through tf-LC3 demonstrated an impairment of the flux primarily at the maturation stage of autophagy/mitophagy in the burn-treated group. Measurement of ROS levels via staining in control and burn groups, with or without EMPA treatment, unveiled a substantial elevation of ROS only in cells subjected to burn serum-stress and subsequent CCCP challenge, exhibiting a remarkable 171.2% increase (p< 0.0001). EMPA treatment effectively attenuated ROS production by 35.0% (p< 0.0001), implying its potential to alleviate stagnated mitophagy flux.

**Conclusions:**

SGLT2 inhibitors like empagliflozin show promise beyond diabetes management. Our study introduces novel methods to assess mitophagy and evaluate EMPA’s efficacy in mitigating mitophagy defects in burn-related diseases, especially critical illnesses, offering potential therapeutic avenues.

**Applicability of Research to Practice:**

Our research on SGLT2 inhibitors like empagliflozin (EMPA) suggests new therapeutic options for mitochondrial dysfunction in burn injuries, potentially improving recovery. The methods can also apply to other disease models, and assessing mitophagy and reactive oxygen species could lead to biomarkers for early diagnosis.

**Funding for the Study:**

Shriners Grant #85106

Hyogo Medical University Fellowship

